# Transdermal delivery of nobiletin using ionic liquids

**DOI:** 10.1038/s41598-019-56731-1

**Published:** 2019-12-27

**Authors:** Tadashi Hattori, Hiroki Tagawa, Makoto Inai, Toshiyuki Kan, Shin-ichiro Kimura, Shigeru Itai, Samir Mitragotri, Yasunori Iwao

**Affiliations:** 1 Laboratory of Pharmaceutical Engineering and Drug Delivery Science, University of Shizuoka, 52-1 Yada, Suruga-ku, Shizuoka, 422-8526 Japan; 2 Laboratory of Synthetic Organic & Medicinal Chemistry, School of Pharmaceutical Sciences, University of Shizuoka, 52-1 Yada, Suruga-ku, Shizuoka, 422-8526 Japan; 3 School of Engineering and Applied Sciences, Harvard University, Cambridge, MA 02138 United States

**Keywords:** Permeation and transport, Biomedical engineering, Biophysical chemistry, Drug development

## Abstract

Nobiletin (NOB), a flavonoid, has extremely low water solubility and low oral bioavailability; however, despite these problems, various physiological effects have been investigated *in vitro*. In the present study, we investigated the transdermal delivery of NOB using choline and geranic acid (CAGE), which is a biocompatible material that has been reported to be a promising transdermal delivery approach. The feasibility was evaluated by a set of *in vitro* and *in vivo* tests. A solubility evaluation demonstrated that CAGE induced excellent solubility of NOB induced by multipoint hydrogen bonding between NOB and CAGE. *In vitro* transdermal tests using a Franz diffusion cell showed that CAGE was effective in enhancing transdermal absorption of NOB, compared to other penetration enhancers. Subsequent *in vivo* tests demonstrated that CAGE significantly improved area under the concentration-time curve of NOB *in vivo* and NOB/CAGE sample showed 20-times higher bioavailability than oral administration of NOB crystal. Furthermore, NOB/CAGE sample also showed significant drops of the blood glucose level in rats derived from hypoglycemic activity of NOB. Thus, transdermal administration of NOB using CAGE was shown to be feasible, which indicates that the use of CAGE may be adapted for other flavonoids that also show both low water solubility and low permeability.

## Introduction

Over the last few decades, the development of combinatorial chemistry and high throughput screening has enabled a large numbers of drug candidates. However, these candidates tend to have large molecular weights and high Log*P* values, which indicates that these compounds are likely to be poorly water-soluble^1^. Greater molecular weight also leads to lower permeability through biological barriers^2^. According to the Biopharmaceutics Classification System (BCS), drugs are classified into four groups: high solubility–high permeability (class I); low solubility–high permeability (class II); high solubility–low permeability (class III); and low solubility–low permeability (class IV)^3^. In particular, to improve the bioavailability (BA) of class IV drugs, the best solution is to go back to the lead optimization phase of drug discovery and modify the structure to obtain the desired physicochemical properties. However, this solution is challenging, time-consuming and costly process. As a result, very few of the millions of compounds tested reach the market. Hence, an alternative option to going back to the lead optimization phase is desirable. Proper formulation is critical to establish successful products for the administration of BCS class IV drugs^4^.

**Figure 1:**
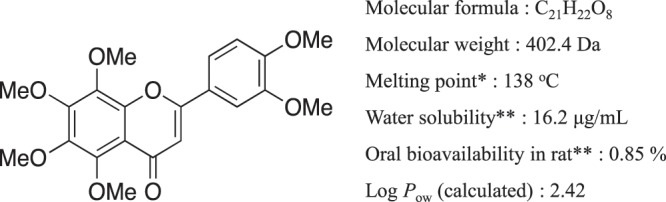
Chemical structure and physicochemical properties of NOB. ^*^Chen *et al*.^18^, **Onoue *et al*.^11^.

Flavonoids are a group of phytochemicals that have shown numerous health benefits and have therefore been studied extensively^5^. However, the low BA of flavonoids, because of phase 2 metabolism^6^ and intestinal first-pass metabolism^7^, is of concern for drug development^5^. In addition, flavonoids tend to have high lipophilicity^8^ derived from the flavone skeletal structure, which leads poor water solubility. Because of these characteristics, flavonoids are categorized as BCS class IV drugs. Nobiletin (NOB) is a flavonoid isolated from the peel of citrus fruits (Fig. 1), which has various reported physiological effects, including anti-Alzheimer’s disease (AD)^9,10^, anti-inflammatory^11^, anti-cancer^12^, and anti-diabetic^13^ effects. However, the solubility of NOB is extremely poor at 16.2 μg/mL^14^ and the BA is also extremely low, 0.85%^14^ for the oral administration of NOB crystals to rats; hence, NOB is classified as a BCS class IV compound. Although many techniques such as solid dispersion^14,15^, self-emulsifying emulsion^16^, nanoparticles^17^, and self-assembled proliposomes^18^ have been used with NOB in attempts to improve the oral absorption, a definitive improvement has not been achieved yet. Thus, a brand- new approach is needed.

In this study, we focused on the transdermal administration of NOB considering the following two points. The first is that the physicochemical properties of NOB are more applicable to transdermal absorption compared with oral absorption. A potential substance will be highly applicable for transdermal absorption according to the following three rules: a molecular weight <500 Da; a melting point <200 °C; and a log*P*_ow_ value from –1 to 4^19,20^. NOB has ideal properties for transdermal absorption with molecular weight, 402.40 Da; melting point, 138 °C^21^; and log*P*_ow_, 2.42 (Fig. 1**)**. The second point considers the possible therapeutic use of NOB. According to a previous study, NOB prevented the hepatic lipid load and consequently limited lipid availability for hepatic storage and lipoprotein secretion and deposition in peripheral tissues, which suggests that NOB has the potential to treat diabetes^22,23^. In the treatment of diabetes, medications are available only through oral or intraperitoneal injection routes. Transdermal administration would provide a valuable option for treatment with easy administration without pain, and consistent drug concentrations in the blood over long periods. There is a need for improved drug delivery system for antidiabetic agents^24^.

In transdermal administration, the impermeability of the outer layer of skin, the stratum corneum (SC), which protects the human body from external pathogens and the evaporation of moisture, creates a formidable barrier to the permeation of large drugs^25–27^. To overcome this barrier, it is important to choose optimal penetration enhancers (PEs) that promote effective absorption and have low skin irritancy. To date, a large number of PEs, including surfactants, fatty acids, terpenes, and other solvents have been reported^28^. Ionic liquids and deep eutectic solvents [hereafter, collectively referred to as ionic liquids (ILs) focusing on their properties as described in the previous report^29^] have gained recent attention as novel enhancers^30^. Generally, ILs are molten salts composed of cations and anions^31^ and the use of many ILs as PEs has been reported^32–34^. Choline and geranic acid (CAGE) is one of ILs^35^. CAGE has been reported to be an excellent PE for various drugs with small^35^ to large^36,37^ molecular weights, which does not cause large irritation to the skin^35^. However, it remains unclear whether the use of CAGE is also applicable to flavonoids.

Herein, we demonstrate the use of CAGE for the transdermal administration of NOB. The poor water solubility of NOB was improved by CAGE, because of the multipoint interactions between NOB and CAGE as determined by NMR analysis. In addition, the absorption of NOB into the skin was significantly improved non-invasively using CAGE. Moreover, the administration of NOB with CAGE significantly increased the area under the concentration-time curve (*AUC*_0-1440min_) of NOB *in vivo*, and showed a positive effect on blood glucose levels, in rats. This knowledge will provide a new option when considering the formulation of flavonoids.

## Results

### Evaluation of NOB solubility in CAGE and the interaction between NOB and CAGE

**Figure 2:**
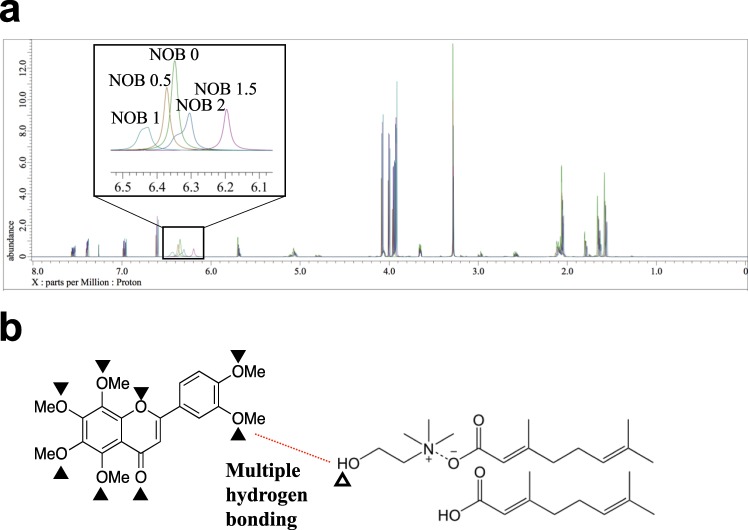
Overlapped ^1^H NMR spectra of NOB-CAGE complexes at each molar ratio (**a**) and a schematic diagram of the interactions between NOB and CAGE (**b**). NOB was mixed with CAGE at different molar ratios [CAGE: NOB = 1:0 (NOB 0), 2:1 (NOB 0.5), 1:1 (NOB 1), 1:1.5 (NOB 1.5), and 1: 2 (NOB 2) mol/mol]. The mixtures were dissolved with chloroform-*d* and then analyzed by ^1^H NMR. The closed triangles show the O atoms in NOB that might interact with CAGE as hydrogen bonding acceptors. The white triangle shows the H atom in CAGE that might interact with NOB as a hydrogen bonding donor.

Previous reports have demonstrated that ILs and DESs have the ability to improve the solubility of poorly water-soluble drugs^38,39^. Therefore, the solubility of NOB in CAGE or water was evaluated to investigate whether CAGE also improved the solubility of NOB. The solubility of NOB in CAGE was 7.2 mg/mL, whereas that of NOB in water is reported to be 0.016 mg/mL (Table 1), indicating that the solubility of NOB was remarkably increased in CAGE by approximately 450 times compared with water. To elucidate the reason why the solubility was enhanced in NOB/CAGE, the mixtures which had been prepared at different molar ratios [CAGE: NOB = 1:0 (NOB 0), 2:1 (NOB 0.5), 1:1 (NOB 1), 1:1.5 (NOB 1.5), and 1:2 (NOB 2)] were each analyzed by ^1^H NMR. Almost all the NMR spectra for each of the samples were well overlapped; however, differences were observed in the spectra from 6.1–6.5 ppm when the molar ratio of the mixture was changed (Fig. 2). This result indicated that NOB interacted with CAGE by hydrogen bondings^40,41^; that is, the H atom of the hydroxyl group in CAGE (6.1–6.5 ppm in NMR spectra) acts as a hydrogen bonding donor, and the O atoms of the carbonyl and methoxy groups in NOB might act as multipoint hydrogen bonding acceptors. These results appear to be in good agreement with the NOB crystal structure^42^. Therefore, it is possible that the hydrogen bondings between NOB and CAGE contributed to the improvement in the solubility of NOB.

**Table 1:** Solubility of NOB in water and in CAGE.

Sample	Solubility (mg/mL)
NOB/water	0.016*
NOB/CAGE	7.200 ± 0.174

^*^Onoue *et al*.^11^.

### Evaluation of the transdermal NOB absorption from NOB/CAGE *in vitro*

**Figure 3:**
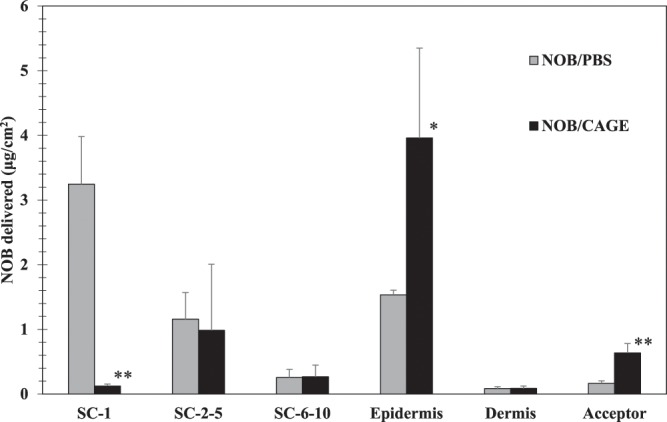
Evaluation of the transdermal absorption of NOB/PBS and NOB/CAGE. Thawed porcine skin without any adipose tissue was fixed with a FDC and 300 μL of 2 mg/mL NOB/PBS or NOB/CAGE were added to the donor of the FDC and incubated for 24 h at 37 °C. After incubation, tape stripping was performed using ten tapes to get parts of the SC (SC1, SC 2–5, and SC 6–10). The remaining epidermis was scraped with a razor and separated from the dermis. The amount of NOB in the SC, epidermis, dermis, and acceptor was quantitatively determined by HPLC as described in Section 2.1.2. Each column represents the mean ± S.D. (*n* = 3). For each fraction, NOB/PBS and NOB/CAGE were compared using the unpaired *t* test. *And **indicate significant differences (*P < 0.05, **P < 0.01).

To evaluate the transdermal absorption of NOB/CAGE *in vitro*, transdermal tests were performed after 24 h incubation using a FDC combined with Yucatan micropig skin (Fig. 3). NOB/PBS (1 mg/mL) in which NOB was suspended in PBS, or NOB/CAGE (1 mg/mL) in which NOB was dissolved in CAGE, were applied to the porcine skin. For NOB/PBS, the amount of NOB detected in SC-1, the fraction of skin surface, was significantly greater than that of NOB/CAGE (*P* < 0.01). In contrast, as for NOB/CAGE, the amount of NOB detected in epidermis and acceptor, deep fractions of the skin, was significantly greater than that of NOB/PBS (*P* < 0.05 or *P* < 0.01). These results indicated that the NOB from NOB/CAGE samples can cross the SC layer without stagnation, and pass through to the deep sites in the skin; while PBS cannot deliver NOB across the SC and the NOB remained at the skin surface. Therefore, NOB absorption and permeation were found to be improved using CAGE.

### Effect of the NOB concentration in PBS or CAGE on the transdermal absorption

**Figure 4:**
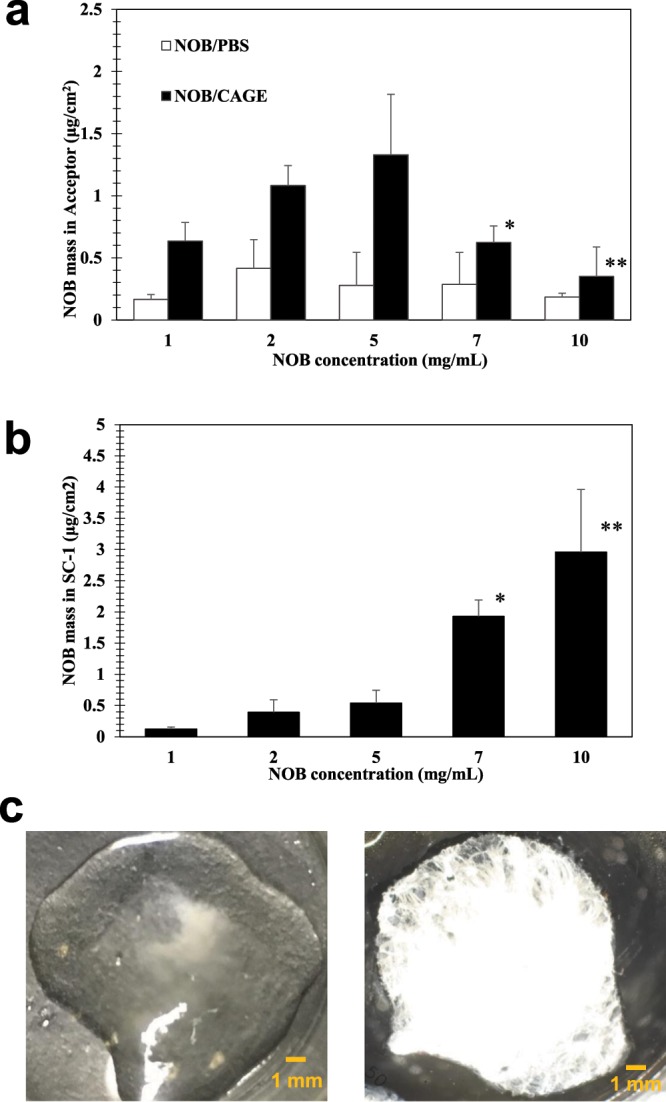
Effect of the NOB concentration in CAGE on the NOB permeation from the skin. (**a)** The amount of NOB detected in the acceptor for each concentration of NOB/PBS and NOB/CAGE. (**b)** The amount of NOB detected in the SC-1 fraction for each concentration of NOB/CAGE. **c)** The images of a donor sample of 7 mg/mL NOB/CAGE in FDC after 24 h incubation taken by a camera (left) and by a polarization microscope (right). For both **a)** and **b)**, each column represents the mean ± S.D. (*n* = 3). The inter-group comparison was carried out using the non-repeated measures ANOVA followed by the Bonferroni test. *and **Indicate a significant difference (*P < 0.05, **P < 0.01) compared with (**a)** 5 mg/mL NOB/CAGE or (**b)** 5 mg/mL NOB/CAGE.

Subsequently, to evaluate in more detail the permeation mechanism of NOB in PBS or CAGE, further transdermal tests were conducted using varied concentrations of NOB in the samples (Fig. 4**)**. For NOB/PBS, there were no significant differences in the amount of NOB detected in the acceptor even if the concentrations were increased. However, for NOB/CAGE, as the NOB concentration increased from 1 to 5 mg/mL, the delivered amount of NOB tended to increase; while interestingly from 5 to 10 mg/mL, the delivered amount of NOB significantly decreased (*P < 0.05 or **P < 0.01) (Fig. 4a**)**. In addition, the amount of NOB detected in SC-1 was increased in a concentration-dependent manner in NOB/CAGE (Fig. 4b). One explanation for this phenomenon is that when there is a high concentration of NOB in CAGE, the NOB may crystalize on the skin surface because water that comes out from the skin interrupts the interaction between NOB and CAGE. Therefore, to investigate this concern, the NOB/CAGE samples (7 mg/mL) before and after incubation were collected and observed using microscopy with a polarization lens. White light emitted from NOB crystals was observed in the sample after incubation, whereas none was observed in the sample before incubation (Fig. 4c). As mentioned above, the solubility of NOB in CAGE was 7.2 mg/mL (Table 1), and at or over this concentration, dissociation of the hydrogen bonds between NOB and CAGE could easily occur. In addition, because of the moisture that came out from the skin, the nucleation and crystal growth of NOB could also occur during incubation, and consequently a decrease in the amount of NOB might be observed. A concentration of <5 mg/mL of NOB in NOB/CAGE was considered to be suitable for further studies.

### NOB permeation with different solvents

**Figure 5:**
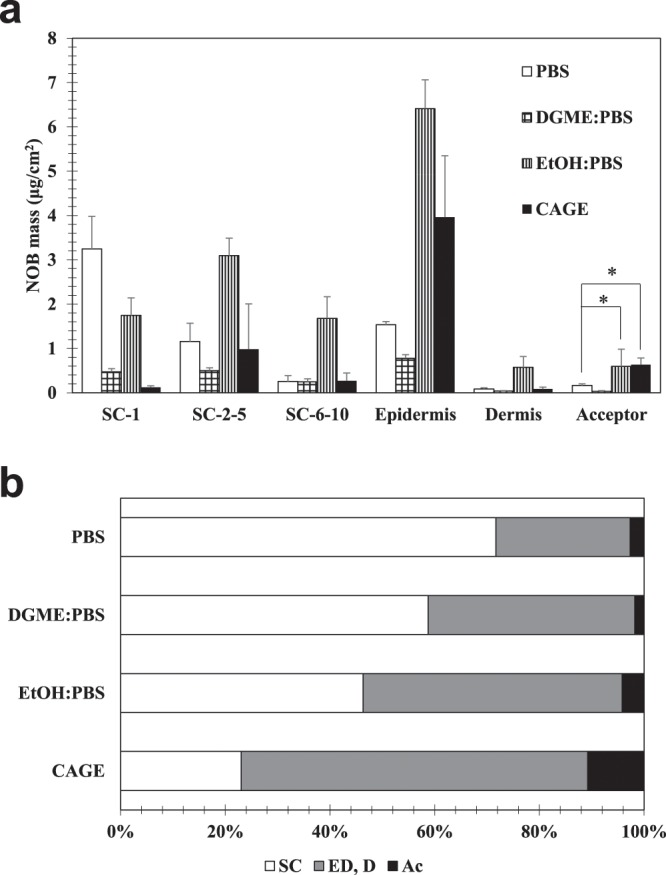
Evaluation of the transdermal absorption of NOB using chemical penetration enhancers. **(a)** Various solutions, 300 μL of 1 mg/mL NOB in PBS, 50:50 (*v*:*v*) DGME: PBS, 50:50 (*v*:*v*) EtOH: PBS, and CAGE were added to the donor of a FDC and incubated for 24 h at 37 °C. After incubation, a tape stripping method was performed using ten tapes to get layers of the SC and the remaining epidermis, dermis, and acceptor were collected to determine the NOB concentration in each fraction. Each column represents the mean ± S.D. (*n* = 3). The inter-group comparison was carried out using the non-repeated measures ANOVA followed by the Bonferroni test. * indicates a significant difference (*P < 0.05) compared with NOB/PBS. (**b**) The ratio of the amount of NOB in each fraction to the total amount. Each column represents the mean (*n* = 3).

To compare the delivery ability of CAGE, additional NOB permeation studies were performed using the widely used chemical penetration enhancers, EtOH and DGME [1 mg/mL NOB in 50:50 (*v*:*v*) DGME: PBS and 50:50 (*v*:*v*) EtOH: PBS]. The amount of NOB detected in the acceptor was in the order CAGE > EtOH: PBS > PBS > DGME and there were significant differences between CAGE and PBS (*P* < 0.05), and PBS and EtOH: PBS (*P* < 0.05) (Fig. 5a**)**. The ratios of NOB detected in the deeper fractions (epidermis + dermis + acceptor) to the total amount of NOB transferred to the skin were CAGE (77%) > EtOH: PBS (54%) > DGME: PBS (41%) > PBS (28%) **(**Fig. 5b**)**. Considering that the amount of NOB detected in the SC fractions was higher with the EtOH: PBS solvent than that with CAGE (Fig. 5a), these results suggested that CAGE has a good ability to deliver NOB across the SC efficiently and specifically.

**Figure 6:**
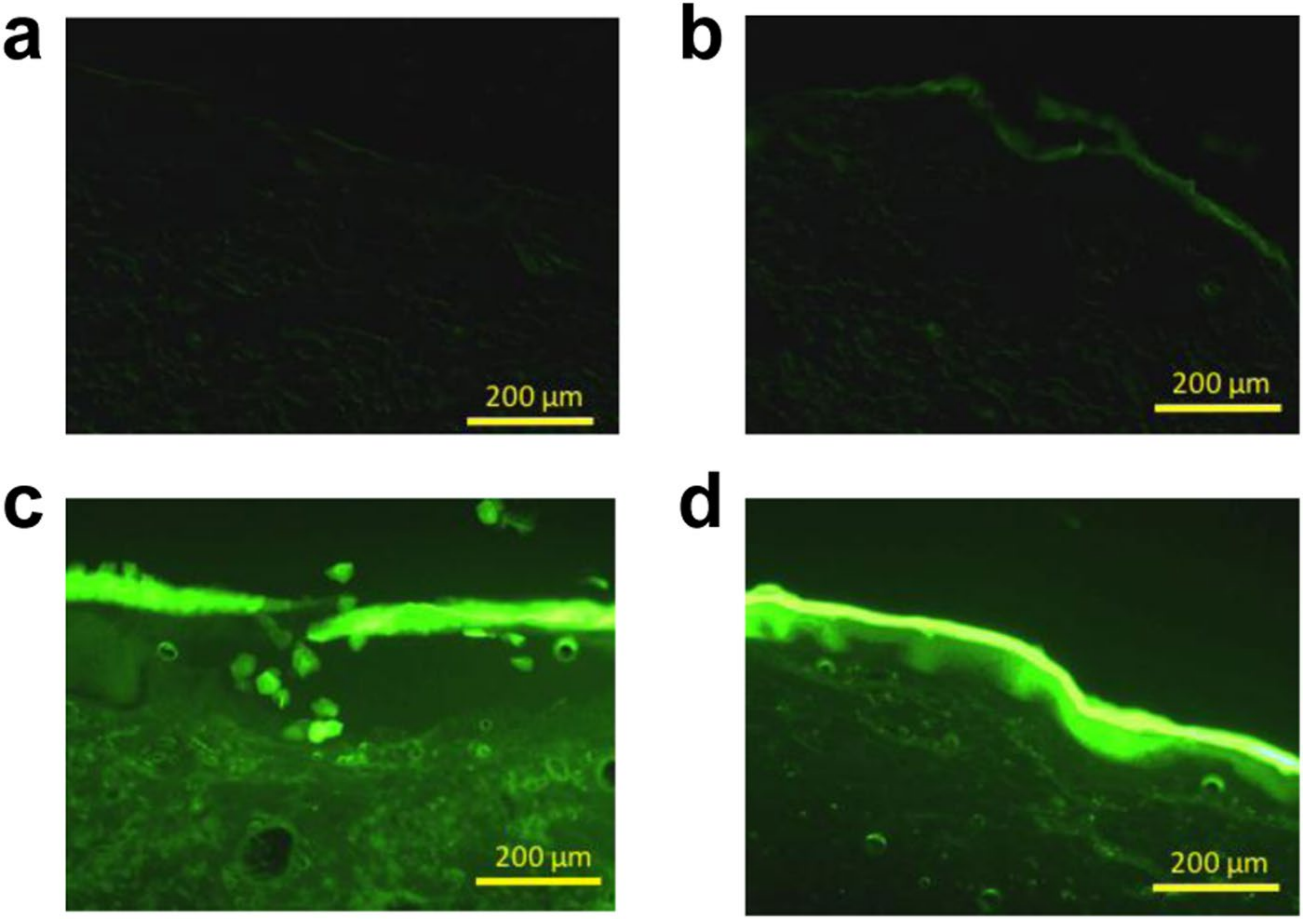
Representative confocal images of skin treated with TG-NOB in (**a**) PBS, (**b**) PBS: DGME, (**c**) PBS: EtOH, and (**d**) CAGE. Each sample was added to the donor of a FDC and incubated for 24 h at 37 °C. After incubation, the skin was soaked in OTC compound in Tissue-Tek^®^ Cryomold^®^ and frozen at −70 °C. The frozen skin samples were sliced to a thickness of 20 μm and then imaged on a fluorescence microscope.

NOB transdermal absorption was visually assessed using fluorescent-labeled NOB (TG-NOB). The samples (1 mg/mL TG-NOB in PBS, 50:50 (*v*:*v*) DGME: PBS, 50:50 (*v*:*v*) EtOH: PBS, and CAGE) were applied to the skin for 24 h, and the skin was then observed using a fluorescence microscope. As shown in Fig. 6, only weak fluorescence was observed in the skin treated with TG-NOB/PBS and TG-NOB/DGME: PBS (Fig. 6a,b); whereas, strong fluorescence was observed in the skin treated with TG-NOB/EtOH: PBS, and TG-NOB/CAGE (Fig. 6c,d**)**. However, the surface of the SC treated with EtOH: PBS was clearly collapsed (Fig. 6c**)**, indicating that EtOH fractured the SC structure as has been previously reported^43^ and this destruction is likely to be involved in the high permeation of NOB into the deep sites of the skin. In contrast, no destruction of the SC was observed with TG-NOB/CAGE (Fig. 6d**)**. Taken together, these results indicate that CAGE can enhance the noninvasive absorption of NOB.

### Evaluation of the NOB transdermal absorption *in vivo*

**Figure 7:**
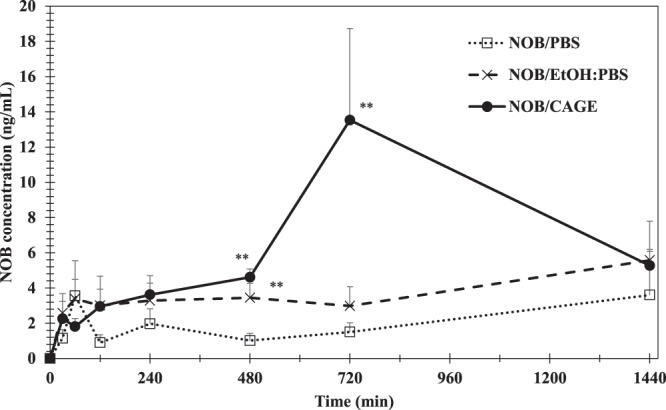
NOB concentration in the plasma after NOB/PBS, NOB/EtOH:PBS, and NOB/CAGE were transdermally administered. Using an electric shaver, the back hair of male SD rats was clipped in a circular shape and a FDC donor was fixed there using instant glue. A 2 mg/mL concentration of NOB/PBS, NOB/EtOH: PBS, or NOB/CAGE was percutaneously administered to the donor area of the rats (5 mg/kg). Each point represents the mean ± S.D. (*n* = 5). The inter-group comparison was carried out using the non-repeated measures ANOVA followed by the Bonferroni test. **Indicates a significant difference (**P < 0.01) compared with NOB/PBS.

Transdermal evaluation of NOB absorption *in vivo* was conducted using SD rats (Fig. 7). PBS, 50:50 (*v*:*v*) EtOH: PBS, and CAGE were selected as test solutions, as DGME: PBS did not enhance NOB absorption *in vitro* **(**Figs. 5 and 6**)**. As for NOB/PBS, the NOB concentration peaked at 60 min after transdermal administration, followed by a slightly decrease and then a stable concentration until 1440 min. For NOB/EtOH: PBS, the NOB concentration increased up to 60 min and then was constant. In contrast, for NOB/CAGE, the concentration increased up to 720 min, followed by a slow decrease to the end point of the study. The values of the pharmacokinetic parameters: maximal concentration (*C*_max_); time for maximal concentration (*T*_max_); and *AUC*_0-1440min_; absolute BA are shown in Table 2. In particular, the values for the *AUC*_0-1440min_ of NOB/PBS, NOB/EtOH: PBS, and NOB/CAGE calculated by the linear trapezoidal method were 2.89 ± 0.91, 5.35 ± 1.43, and 10.57 ± 2.50 μg/mL•min, respectively, and the value for NOB/CAGE was approximately 1.9-fold and 3.7-fold higher compared with the others. Moreover, absolute BAs of NOB/PBS, NOB/EtOH:PBS, and NOB/CAGE were calculated to be 2.47%, 4.58%, and 9.04% respectively. These results suggested that the absorption of NOB was improved using CAGE compared with the other enhancers *in vivo*.

**Table 2:** Pharmacokinetic parameters of NOB.

	NOB/PBS	NOB/EtOH:PBS	NOB/CAGE	NOB solution (*i*.*v*.)
*C*_max_ (ng/mL)	4.85 ± 2.24	7.52 ± 1.96	14.5 ± 4.81	—
*T*_max_ (min)	864 ± 247	889 ± 338	768 ± 192	—
*AUC*_0-1440min_ (μg/mL•min)	2.89 ± 0.91	5.35 ± 1.43	10.57 ± 2.50**	23.37 ± 6.77
Absolute BA (%)	2.47	4.58	9.04	—

Mean ± S.E. (*n* = 5). The inter-group comparison was carried out using the non-repeated measures ANOVA followed by the Bonferroni test. **Indicates a significant difference (**P < 0.01) compared with NOB/PBS.

### Hypoglycemic activity of the NOB/CAGE sample transdermally administrated to SD rats

**Figure 8:**
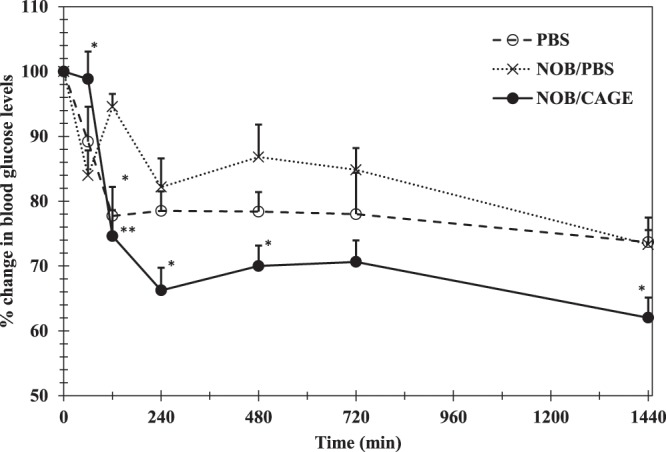
Changes in the blood glucose levels after NOB/PBS, NOB/EtOH: PBS, and NOB/CAGE were transdermally administered. A 5 mg/mL concentration of PBS, NOB/PBS, or NOB/CAGE was transdermally administered (10 mg/kg) to rats and blood was collected from the tail vein up to 24 h. Blood glucose was measured using a commercial glucose meter. Each point represents the mean ± S.D. (*n* = 5). Inter-group comparison was carried out by the non-repeated measures ANOVA followed by the SNK test. * and ** represent a significant difference (*P < 0.05, **P < 0.01) compared to NOB/PBS.

Finally, the transdermal hypoglycemic activity of different samples of NOB was tested using SD rats. The samples were PBS, NOB/PBS, and NOB/CAGE (Fig. 8). A 22% drop in the blood glucose level (BGL) was observed at 120 min after PBS administration. After that, the level remained constant until 1440 min. Approximate drops of 10–20% in the BGL were observed until 720 min after NOB/PBS administration, then a 27% decrease was observed over 720–1440 min. Using NOB/CAGE, a significant 34% drop in the BGL was observed from 0–240 min (*P* < 0.05). Then, the BGL increased slightly followed by a marked 38% decrease from 720–1440 min. These data demonstrated that after NOB/CAGE was administrated, NOB was transdermally absorbed and can elicit pharmacological effects, such as hypoglycemic activity, *in vivo*.

## Discussion

In this study, we investigated the feasibility of the transdermal absorption of the polymethoxy flavonoid, NOB, which has extremely poor water solubility and poor absorption, using the enhancer CAGE. CAGE demonstrated an ability to transdermally transport NOB to the deeper sites of the skin and enhanced the permeation of NOB *in vivo*.

When NOB was mixed with CAGE, the solubility of NOB was improved by approximately 500 times. NMR analysis indicated that the H atom of the hydroxyl group in CAGE was a hydrogen bonding donor and the two O atoms in the skeleton of the flavonoid, or all seven methoxy groups in NOB, appeared to be hydrogen bonding acceptors. This result was supported by the results of X ray crystal structural analysis^42^. Generally, the skeletal structures of flavonoids hinder their dissolution in water through strong π-π interactions. It has been reported that an OH or OCH_3_ substituent on ring B of a flavonoid, the ring B connection position, the bond order between C_2_ and C_3_ (single or double), and the presence of an OH substituent at the 3-position can influence the solubility of flavonoids^44^. However, all flavonoids have at least one O atom in the skeletal structure, indicating that CAGE could improve the solubility of flavonoids other than NOB. In particular, improvements in compounds that are poorly water-soluble and have poor oral BA, such as quercetin^45,46^ (solubility in water: 0.0014 mg/mL, BA in men: 1%) and kaempferol^8^ (solubility in water: 0.18 mg/mL, BA 2%) could be expected.

In the transdermal absorption studies *in vitro*, CAGE enabled NOB to permeate into deep site of the skin without stagnation in SC fractions. Similar phenomenon was observed in a previous report by Banerjee *et al*.^37^, in which CAGE enhanced the transdermal absorption of other compounds with a large molecular weight, including fluorescein isothiocyanate labelled insulin and fluorescein isothiocyanate labelled bovine serum albumin. The use of CAGE improved the noninvasive absorption of NOB in comparison with other PEs. The present study also confirmed that EtOH can cause skin irritation. Irritation caused by EtOH has been previously reported. Farkas *et al*. have reported that EtOH induced proliferation of HaCaT cells, which could induce psoriasis^43^. Goates *et al*. have reported that EtOH extracted, not only SC lipids, but also SC proteins, by changing their conformation^47^. On the other hand, it has been reported that CAGE extracted only lipids^35^. These differences in the effects on the SC led to differences in the permeability between CAGE and EtOH. The mechanism of EtOH permeation, which causes conformational alterations in the SC proteins, was suggested that EtOH delivered NOB to a wide area of the SC, which meant that NOB tended to remain in the SC fractions. Therefore, this mechanism of EtOH permeation led to unselective delivery, as shown in in Fig. 5b, as well as causing irritation. In contrast, CAGE only had an effect on lipids, which meant that CAGE had less of an effect on the SC, compared with EtOH. Because CAGE causes only small changes in the SC structure, CAGE may be useful for noninvasive and selective deep-skin delivery. However, further studies regarding the mechanism of how CAGE has a permeation enhancing effect without causing irritation to the skin are needed.

*In vivo*, CAGE improved transdermal NOB absorption and had a plasma concentration profile that was different compared with the other enhancers tested. A large increase in the plasma concentration of NOB was observed at 720 min. This increase may be caused by the way NOB penetrated into the deep skin along with CAGE. Banerjee *et al*. have reported that delivered fraction of ^3^H BSA in the skin greatly changed from the SC to the epidermis, dermis, and acceptor between 12 and 24 h^37^. In the present study, the NOB concentration was increased at 12 h and the rate of NOB penetration appeared to be fast. These different results might be explained by differences in the skin structure of rat and pigs; rat skin is thinner compared with the pig skin used in previous work^48^. NOB penetrated into skin layers deeper than the SC after approximately 12 h, which led to the observed increase in the NOB concentration at that time. Also, a decrease in the plasma concentration of NOB was observed at 1440 min. This decrease might be because NOB was removed from the blood through metabolism and excretion, and because the amount of NOB transferred into the blood decreased because the skin was filled with CAGE and NOB. Moreover, absolute BAs were calculated. Onoue *et al*. reported that the oral BA of crystalline NOB (2 mg/kg) was 0.46%^15^. In this study, the BA of NOB/CAGE (9.04%) was approximately 20-times higher than oral administration of NOB crystalline and even NOB/PBS marked more than five times BA than that of crystalline NOB. This is because avoiding first pass metabolism of NOB by means of transdermal administration. In this test, 2 mg/mL sample was used and the duration of the test was 24 h. Figure 4(a) demonstrated NOB amount of that permeated through skin increased concentration-dependent manner up to 5 mg/mL. Thus, optimization of concentration of the sample could give even greater bioavailability.

Furthermore, transdermal hypoglycemic activity was evaluated with nondiabetic rats. Interestingly, transdermal administration of NOB using CAGE decreased the BGL, whereas there was no significant difference between PBS and NOB/PBS. These results suggested that CAGE transported a sufficient amount of NOB into the blood to decrease the BGL, whereas PBS did not. Although the PBS sample showed a larger decrease in BGL than NOB/PBS, this might be because of prolonged fasting^49^. It has been reported that oral administration of 200 mg/kg NOB significantly suppressed weight gain and caused a drop in the BGL accompanied with insulin injection^49^. In the present study, transdermal administration of only 10 mg/kg NOB alone caused a significant decrease in the BGL (*P* < 0.05 or 0.01). This result might be because the transdermal administration prevented NOB from being metabolized by the first pass effect, which made the expression of the hypoglycemic action of NOB more efficient. Transdermal insulin delivery using CAGE has been shown to induce almost a 40% drop in the BGL over 12 h^37^. In contrast, although NOB induced a relatively small decrease in the BGL (at most 34%) over 12 h in the present study, this indicated that transdermal administration of NOB may avoid excessive hypoglycemia. Thus, NOB is a potential new therapeutic, or preventive ingredient, for diabetes that has less risk of side effects, such as hypoglycemia.

## Conclusion

NOB has extremely low water solubility and low oral bioavailability, and a brand-new approach to solve these concerns is needed to show various physiological effects of NOB *in vivo*. In this study, we investigated the feasibility of the transdermal absorption of NOB, using an ionic liquid, CAGE. CAGE induced excellent solubility of NOB induced by multipoint hydrogen bonding between NOB and CAGE. In addition, CAGE was found to be effective in enhancing transdermal absorption of NOB without invasiveness by comparing to other penetration enhancers. Furthermore, after NOB/CAGE was transdermally administrated, NOB bioavailability was 20-times higher than oral administration of NOB crystal and NOB/CAGE significantly reduced the blood glucose level in rats derived from hypoglycemic activity of NOB. Therefore, feasibility of transdermal absorption of NOB with CAGE was confirmed, indicating that wider use of this transdermal absorption system using CAGE against other flavonoids as well as BCS class IV drugs would be expected.

## Materials and Methods

### Materials and animals

NOB was synthesized by an established method^50^ and provided by the Ushio Chemix Corp. (Shizuoka, Japan). Geranic acid (85%), choline bicarbonate (~80% in water), dimethyl sulfoxide-*d*_6_ (99.5 atom %D), chloroform-*d* (99.8 atom % D), and phosphate buffered saline tablets were purchased from Merck KGaA (Darmstadt, Germany). Acetone, 4% paraformaldehyde phosphate buffer solution, formic acid, and ethanol were purchased from Wako Pure Chemical Industries (Osaka, Japan). Diethylene glycol ethyl methyl ether was purchased from Tokyo Chemical Industries (Tokyo, Japan). Porcine skin (Yucatan Micropig) was purchased from Charles River Laboratories International (Yokohama, Japan). All other chemicals were of the highest grade commercially available, and all solutions were prepared in deionized and distilled water.

### Methods

#### Preparation of CAGE

CAGE was prepared according to an established method^35^. Two equivalents of technical grade 85% geranic acid (5.07 g, 0.0301 mol) was recrystallized at −70 °C from 70 wt% geranic acid/30 wt% acetone. To this geranic acid in a 100 mL eggplant flask was added one equivalent of choline bicarbonate (80 wt% solution, 3.109 g, 0.0151 mol). The mixture was stirred at room temperature until no more CO_2_ evolved. The solvent was removed by rotary evaporation at 60 °C for 20 min, and the product was dried in a vacuum oven for 48 h at 60 °C to give CAGE (6.583 g, 0.0150 mol). The NMR assignments (using a JEOL ECX500) were in good agreement with a previous study^35^: ^1^H NMR (dimethyl sulfoxide-*d*_6_), δ5.58 (s, 2H), 5.06 (t, J = 6.9, 2H), 3.85 (m, J, 2H), 3.43 (m, 2H), 3.12 (s, 9H), 2.54 (m, 4H), 2.06 (m, 4H), 1.99 (m, 6H), 1.73 (s, 2H), 1.64 (s, 6H), and 1.56 (s, 6H); ^13^C NMR (DMSO- *d*_6_), δ 170.0, 149.7, 131.1, 123.7, 121.5, 67.2, 55.1, 53.1, 53.1, 32.4, 25.8, and 17.7.

#### Measurement of NOB solubility in CAGE

An excess amount of NOB was added to CAGE in test tubes and the tubes were shaken at 37 °C for 48 h. The supernatants were then collected and filtered through a filter with a pore size of 0.20 µm (Toyo Roshi Co. Ltd., Tokyo, Japan), and the concentration of NOB was determined by high-pressure liquid chromatography (HPLC) (Shimadzu LC-2010C, Kyoto, Japan) with the following conditions: mobile phase 0.1% formic acid in water (solvent A) and acetonitrile (solvent B) (0–20 min, 52.5–75% solvent A); flow rate 1 mL/min; injection volume 5 µL; column Develosil ODS-HG-5, 4.6 mm × 150 mm, 5 µm particle size; column temperature 40 °C; UV detector wavelength 285 nm.

#### Analysis of NOB–CAGE interaction using ^1^H NMR

NOB and CAGE were mixed at molar ratios of 1:0 (0.05:0 mol), 2:1 (0.05:0.025 mol), 1:1 (0.05:0.05 mol), 1:1.5 (0.05:0.075 mol), and 1:2 (0.05:0.01 mol). Each mixture was dissolved in chloroform-*d* and then analyzed by ^1^H NMR.

### *In vitro* skin penetration test using Franz diffusion cell

Porcine skin stored at −70 °C was thawed at 37 °C for 30 min, and then cut into a circle 3.5 cm in diameter. The adipose tissue of this skin was scraped off with a razor. The skin was sandwiched between the acceptor side of a Franz diffusion cell (FDC) (PermeGear, Hellertown, PA, USA) filled with phosphate buffered saline (PBS) solution, and the donor of the FDC, and both sides of the FDC were fixed with a clip. The skin was stretched without wrinkles and the acceptor was degassed. Then, 300 μL of 1, 2, 5, 7, and 10 mg/mL NOB/PBS or NOB/CAGE were added to the donor and incubated for 24 h at 37 °C. After incubation, samples in the donor were removed and the skin was washed up to 5 times with PBS solution. The skin was removed from the FDC and punched out according to the administration area. A tape stripping method was used with ten tapes to obtain parts of the stratum corneum (SC). The remaining epidermis was scraped with a razor and separated from the dermis. NOB was eluted from these tissue samples using methanol: PBS = 1:1 for 24 h. The solutions were centrifuged at 3,000 rpm at 25 °C for 3 min and the supernatant was passed through a membrane filter (pore size: 0.20 µm, Toyo Roshi Co. Ltd.). The amount of NOB in the SC, epidermis, dermis, and acceptor was quantitatively determined by HPLC as described in Section 2.1.2.

In addition, when the transdermal tests using 10 mg/mL NOB/CAGE incubated for 24 h were completed, the NOB/CAGE sample in the donor of the FDC was collected and observed using a polarizing microscope (OLYMPUS SZ 61, Olympus Corporation, Tokyo, Japan) to check the sample condition.

To compare the permeation ability of CAGE with other skin permeation reagents, additional 24-h transdermal tests were performed using 1 mg/mL NOB/diethylene glycol monoethyl ether (DGME): PBS = 1:1 (*v:v*) and NOB/ethanol (EtOH): PBS = 1:1 (*v:v*), in addition to NOB/PBS and NOB/CAGE.

### Observation of the transdermal absorption of NOB using Tokyo Green labeled-NOB (TG-NOB)

To visually observe the transdermal absorption of NOB, NOB labeled with a green fluorescent dye, Tokyo Green^51^ was used. A transdermal penetration study was performed using 1 mg/mL TG-NOB in PBS, 50:50 (*v*/*v*) DGME: PBS, 50:50 (*v*/*v*) EtOH: PBS, or CAGE incubated for 24 h following the same protocol as in Section 2.2. After incubation, the skin was washed 5 times with PBS and cut by a medical scalpel to a thickness of 3 mm. The skin was soaked in optimal cutting temperature (OTC) compound (Sakura Finetek Japan Co., Ltd. Tokyo, Japan) in Tissue-Tek^®^ Cryomold^®^ (Sakura Finetek Japan Co., Ltd. Tokyo, Japan) and frozen at −70 °C. The frozen skin sample was sliced to a thickness of 20 μm using Cryostat (LEICA CM 3050 S, Leica Biosystems, Nussloch, Germany) and fixed to a glass slide. This glass slide was immersed in a 4% paraformaldehyde PBS solution for 15 min and washed with PBS, then imaged on a fluorescence microscope (OLYMPUS IX 71, Olympus Corporation, Tokyo, Japan).

### *In vivo* transdermal evaluation using SD rats

Male Sprague-Dawley (SD) rats (weight: 280–300 g, age: 8–9 weeks; Japan SLC, Shizuoka, Japan) were fasted overnight for 8 h prior to the experiment and given access to water *ad libitum*. All of the procedures used in this study were performed in accordance with the guidelines approved by the Institutional Animal Care and Ethical Committee of the University of Shizuoka, Japan, as described previously^52,53^. Using an electric shaver (5030 s, The Procter & Gamble Company of Japan Limited, Hyogo, Japan), the back hair was removed in a circular shape (3 cm in diameter) and a Franz cell donor was fixed there using instant glue. In order to track plasma concentration of NOB administrated transdermally, a 2 mg/mL concentration of NOB/PBS, NOB/EtOH: PBS = 1:1 (*v:v*), or NOB/CAGE was percutaneously administered to the donor area of the rats (5 mg/kg) and blood was collected from the tail vein at 30, 60, 120, 240, 480, 720, and 1440 min. (n = 5) Moreover, 1 mg/mL NOB solution was intravenously injected (i.v.) for calculating absolute BA. The solution was prepared by mixing 2% Tween 80 aqueous solution, NOB and dimethyl sulfoxide. The blood was collected from the tail vein at 1, 3, 5, 8, 10, 30, 45, 60, 90, 120, 240, 360, 480, 720, 1440 min. (n = 3) The blood samples were centrifuged at 10,000 rpm, 4 °C, for 10 min, and 100 μL of the obtained supernatant was added to a mixture of 50 μL of 0.5 mM formic acid aqueous solution and 850 μL of methanol, followed by vortexing for 10 min and sonication and centrifugation at 10,000 rpm, 4 °C, for 10 min. The obtained supernatant was filtered through a 0.2 μm filter to obtain a measurement sample. The concentrations of NOB in the rat plasma were determined using an ACQUITY UPLC™ system (Waters, Manchester, UK), equipped with an electrospray ionization (ESI) interface, as described previously^52^. Separation was carried out using a Cadenza CD-C18 column, 3 μm, 4.6 mm × 150 mm (Imtakt). The column was kept at 40 °C. The mobile phases consisted of solvent A: solvent B = 82:18 (*v:v*); solvent A (acetonitrile), and solvent B (water, 0.1% formic acid). The injection volume was 5 μL and the effluent from the column was directly introduced into the ion source of the mass spectrometer without splitting. MS/MS detection was performed using an ACQUITY TQD tandem quadrupole mass spectrometer (Waters Co., Milford, MA, USA). The ESI interface was used in positive ESI (m/z: 403.4 → m/z: 373.4). Data acquisition was performed in multiple reaction monitoring (MRM) mode.

### Confirmation of hypoglycemic activity from NOB/CAGE sample transdermally administrated to SD rats

The hypoglycemic activity of the NOB/CAGE sample was evaluated following the same protocol used for the transdermal tests *in vivo* using SD rats as described in Section 2.4. A 5 mg/mL solution of PBS, NOB/PBS, or NOB/CAGE was transdermally administered to the donor area of the rats (10 mg/kg) and blood was collected from the tail vein after 1, 2, 4, 8, 12, and 24 h. Blood glucose was measured using a commercial glucose meter (ACCU-CHEK^®^ ST Meter, ACCU-CHEK^®^ ST Strip, Roche Diagnostics K.K., Tokyo, Japan) (n = 5).

### Statistics

Statistical analyses were performed by using the Student’s t-test. Statistical analyses for multiple comparisons were determined by the analysis of variance (two-way ANOVA) followed by the Student-Newman-Keuls (SNK) test or Bonferroni analysis, as described previously^54^. Results with *P* < 0.01 or *P* < 0.05 were considered to be statistically significant.
