# Author Correction: Transdermal delivery of nobiletin using ionic liquids

**DOI:** 10.1038/s41598-020-60921-7

**Published:** 2020-02-27

**Authors:** Tadashi Hattori, Hiroki Tagawa, Makoto Inai, Toshiyuki Kan, Shin-Ichiro Kimura, Shigeru Itai, Samir Mitragotri, Yasunori Iwao

**Affiliations:** 1 Laboratory of Pharmaceutical Engineering and Drug Delivery Science, University of Shizuoka, 52-1 Yada, Suruga-ku, Shizuoka 422-8526 Japan; 2 Laboratory of Synthetic Organic & Medicinal Chemistry, School of Pharmaceutical Sciences, University of Shizuoka, 52-1 Yada, Suruga-ku, Shizuoka 422-8526 Japan; 3 School of Engineering and Applied Sciences, Harvard University, Cambridge, MA 02138 United States

Correction to: *Scientific Reports* 10.1038/s41598-019-56731-1, published online 27 December 2019

In this Article, the corresponding author neglected to include some potential conflicts of interest on behalf of all authors. The Competing Interests section should read:

“SM is a shareholder/board/member/consultant of Cage Bio and Liquideon LLC which have licensed the patent on cage (US patent 10,449,254) from University of California, of which SM is an inventor. TH, YI, HT, SK, MI, TK, SI declare no potential conflict of interest”

